# Pluripotent Stem Cells of Order Carnivora: Technical Perspective

**DOI:** 10.3390/ijms24043905

**Published:** 2023-02-15

**Authors:** Aleksei G. Menzorov

**Affiliations:** 1 Sector of Cell Collections, Institute of Cytology and Genetics of the Siberian Branch of the Russian Academy of Sciences, 630090 Novosibirsk, Russia; menzorov@bionet.nsc.ru; 2 Natural Sciences Department, Novosibirsk State University, 630090 Novosibirsk, Russia

**Keywords:** iPS cells, ES cells, reprogramming, Carnivora

## Abstract

Human and mouse induced pluripotent stem cells (PSCs) are widely used for studying early embryonic development and for modeling of human diseases. Derivation and studying of PSCs from model organisms beyond commonly used mice and rats may provide new insights into the modeling and treating human diseases. The order Carnivora representatives possess unique features and are already used for modeling human-related traits. This review focuses on the technical aspects of derivation of the Carnivora species PSCs as well as their characterization. Current data on dog, feline, ferret, and American mink PSCs are summarized.

## 1. Advantages of Carnivora Pluripotent Stem Cells

The majority of the research on pluripotent stem cells (PSCs) focuses on just two organisms—mouse and human. Embryonic stem (ES) cells were first derived from mouse blastocysts [1,2]. Human ES cells were derived by Thomson in 1998 [3]. In 2006, Yamanaka developed a methodology to efficiently reprogram somatic cells into the induced pluripotent stem (iPS) cells [4]. ES and iPS cells are exceptionally similar in gene expression and DNA methylation patterns [5]. Somatic cell reprogramming with transcription factors make it possible to overcome the limitations of the ES cells: the limited number of blastocysts and ethical concerns for the usage of embryonic material. iPS cell technology made possible the production of PSCs from different species including representatives of the order Carnivora.

The popularity of model organisms depends on many factors. The most popular mammalian models are mice and rats, fast-reproducing rodents that allow the modeling of many aspects of human diseases. The main limitation is that, as with any other model organism, mice and rats are not humans. Additional model organisms may provide complex modeling of human diseases, and it is better to use several models for particular purposes. Which Carnivora species may be a good additional model?

The dog is a promising model organism. Dogs live significantly longer than mice and rats and share many diseases with humans. That makes a dog a good model for age-related diseases [6]. The OMIA database (https://www.omia.org/, accessed on 11 November 2022) had 867 genetically controlled features that involved at least 317 genes. That information may facilitate the modeling of different human diseases in dogs.

The cat also has many important features for studying human genome abnormalities. There are many human genome variants that have no known function. Cat homologs may elucidate their significance [7]. Cat genetics is fairly well studied. The OMIA database (https://www.omia.org/, accessed on 11 November 2022) had 401 genetically controlled features that involved at least 95 genes.

Other carnivorans are less studied. For instance, there are just 11 traits in the OMIA database with 2 related genes for ferrets. On the other hand, a ferret is suitable for studying various human pathogens, such as influenza virus, respiratory syncytial virus, measles virus, and others [8]. An important part of the recent pandemic caused by SARS-CoV-2 is animal transmission of the virus. Felines, dogs, ferrets, minks, and other animals tested positive for SARS-CoV-2 [9]. Though virus adaptation to ferrets and minks has not yet provided an advantage regarding human infection [10], that may change in the virus evolution in the future. It may be advantageous to have animal in vitro and in vivo models to study virus variants.

**Figure 1:**
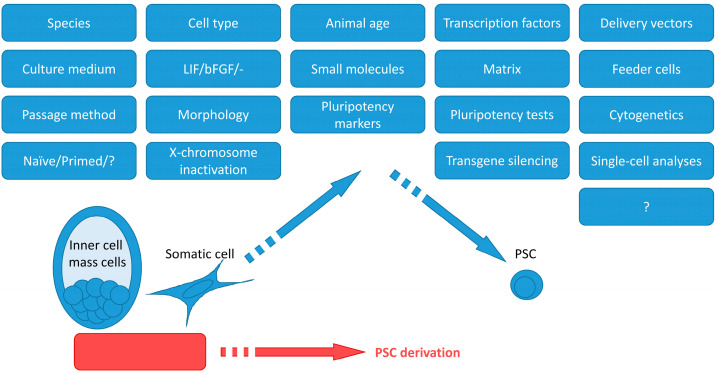
Important factors for the derivation, maintenance, and analysis of PSCs.

Carnivoran PSCs could be used in different ways. First, PSC generation with subsequent differentiation into various cell types in vitro and in vivo is a viable opportunity for modeling human diseases. Second, ES and iPS cells make possible studies of the biology of the species that are in the shadow of the mice and rats. Third, PSCs could be potentially used for endangered species restoration through differentiation into gametes. Such a strategy is in development for the white rhinoceros [11]. This review focuses on the technical aspects of ES and iPS cell derivation and may be useful for planning experiments on PSC derivation from novel species. Table S1 summarizes the current literature on PSC derivation from carnivorans. Figure 1 represents important factors that are necessary to bear in mind when attempting to produce PSCs. PSCs were produced from dogs [12,13,14,15,16,17,18,19,20,21,22,23,24,25,26,27,28,29,30,31], felines [32,33,34], ferrets [35,36], and American mink [37,38]. The articles describing cell lines with unproven pluripotency were excluded from the review.

## 2. PSC Derivation and Culture Conditions

### 2.1. Species and Cell Types

Currently, ES and iPS cells from the order Carnivora were produced from just several species: dog, cat, ferret, American mink, snow leopard, and Bengal tiger (Table S1). Probably, there were unsuccessful attempts to produce iPS cells from the other species, as negative results usually remain unpublished. The majority of the articles cover dog PSCs, as dogs are a companion animal as well as a relatively well-studied model organism. The possible role of the animal genotype will be covered in the Section 3.2.

Carnivora ES cells were produced from blastocysts, the same as for human and mouse. As for the iPS cell derivation, the majority of the researchers used fibroblasts. Fibroblasts are an easily obtainable cell type that is routinely maintained in culture. One of the drawbacks of fibroblast usage is possible contamination with mesenchymal stem cells that have similar morphology and are practically undistinguished by surface markers [39]. Multipotent mesenchymal stem cells might have different reprogramming efficiency than nullipotent fibroblasts. They also have endogenous expression of such pluripotency marker genes as *Oct4*, *Sox2*, and *Rex1* [40,41]. Mesenchymal stem cells could be found throughout the body. Notably, Dutton and colleagues used adipose tissue fibroblasts for cat iPS cell derivation [34]. It is possible that the percentage of mesenchymal stem cells may be higher in this tissue than in other fibroblast sources. Another cell type used for iPS derivation is blood cells [18]; they are readily available, but the efficiency of iPS cell derivation may differ.

### 2.2. Animal Age

Animal age is most probably important for iPS cell derivation. Cell divisions are considered to be necessary for somatic cell reprogramming into pluripotency, and cells from older animals may have lower proliferation potential. It was shown that human donor age negatively influences the efficiency of iPS cell generation though not their properties [42]. It may be a good idea to use embryonic fibroblasts when possible.

### 2.3. Transcription Factors and Delivery Vectors

The majority of Carnivora iPS cells were produced using human transcription factors OCT4, KLF4, SOX2, and MYC. Those transcription factors appear to be evolutionally conservative, and the homology is enough to reprogram somatic cells of mouse and several Carnivora species (Table S1). Other transcription factor sets such as OCT4, SOX2, KLF4, MYCL, LIN28, and mp53DD were also initially developed for human iPS cell derivation. Koh and coauthors used murine transcription factors for dog iPS cell derivation [21]. The decision was probably based on the availability of the retroviral vectors [4]. Probably, the usage of species-specific transcription factors would be beneficial, but that requires additional comparative research.

Originally, retroviral vectors were used to deliver transgenes [4]. There are two major drawbacks of these vectors: they infect only dividing cells and are inserted into the genome. Lentiviral vectors are superior, as they infect cells regardless of the cell cycle. The insertion into the genome is a worse obstacle. On the one hand, PSCs silence lentiviral transgenes [43,44,45], so after the establishment of pluripotency, exogenous transgenes are mostly not expressed. On the other hand, transgenes may reactivate and reduce PSC differentiation capacities [46] and make them prone to malignancy; for instance, MYC is a proto-oncogene [44].

Non-integrating vectors are preferable for PSC derivation. Such vectors include episomes, Sendai-virus-based, adenovirus-based, adeno-associated virus-based, modified RNA, and others. The risk of genome integration of DNA-based vectors exists but overall is negligible. We have successfully tested Sendai-virus-based CytoTune EmGFP Sendai Fluorescence Reporter (Thermo Fisher Scientific, Carlsbad, CA, USA) in pinniped fibroblasts [47]. On day 4, the fluorescence was significantly higher for the Sendai-virus-based vector than for the lentiviral-based one. The use of CRISPR/Cas-based gene expression manipulation or reprogramming with just some small molecules is beyond the scope of this review.

### 2.4. Culture Medium

The majority of the researchers used DMEM/F12 or KnockOut DMEM (KO-DMEM)-based culture medium for Carnivora PSC derivation. Mouse fibroblasts and PSCs are routinely maintained in DMEM and human in DMEM/F12. Probably, that is the reason for the usage of these media for dog and other carnivoran cells. The other option is to use MEM α, as Carnivora fibroblasts are mainly cultured in that medium as well as American mink ES and iPS cells [37,38], but the advantage of that choice should be tested experimentally.

Several research groups successfully used N2B27-based culture media during the initial stages of cell reprogramming [15,16,19,20,31]. Mouse embryonic fibroblasts (MEFs) could be reprogrammed into neural stem cells with Sox2, Klf4, and Myc with a brief expression of Oct4 [48]. Supposedly, iPS cells may be generated more efficiently through the neural stem cell stage.

FBS (fetal bovine serum) is used for maintaining the majority of cell types. As it varies from batch to batch, researchers usually pre-test FBS batches for “demanding” cell types such as mesenchymal stem cells or mouse PSCs. Untested FBS may influence cell proliferation and cause differentiation. It may be surmised that Thomson was able to produce human ES cells, as he had a batch of FBS qualified for monkey ES cells [3,49]. Nowadays, ES cell-qualified FBS exists for mouse PSCs. Still, the fact that FBS is qualified to support the growth of mouse PSCs does not guarantee that it will support the pluripotency of stem cells of different species. About one-third of researchers reported using FBS for Carnivora ES or iPS cell derivation, while others use KnockOut Serum Replacement for feeder-based cell culture or feeder-free, serum-free formulations such as mTeSR1 or StemFit (Table S1). The principal limitation for Carnivora PSCs is that these reagents were developed for human PSC pluripotency support. The culture conditions suitable for human PSCs are not necessarily proper for cells of the other species.

Pluripotency of the naïve mouse and human PSCs depends on leukemia inhibitory factor (LIF) and BMP4 and of the primed PSCs on basic fibroblast growth factor (bFGF) [50]. It seems plausible that Carnivora PSCs would also be dependent on these growth factors. In mouse and human, the signal pathways with LIF and bFGF involvement are mutually exclusive. Thus, either LIF or bFGF is used for cell culture. Interestingly, many researchers used both LIF and bFGF throughout the Carnivora PSC derivation or during the initial steps. On the other hand, Wilcox and colleagues noted that bFGF caused the differentiation of dog ES cells [14]. Such discrepancy in cell culture conditions would be briefly covered in the Section 3.2. Researchers mainly used human or murine LIF and bFGF due to their availability. Contrary to that, Dutton and colleagues showed that for the cat iPS cell derivation and maintenance, only feline LIF could be used, not murine [34]. Another notable exception is American mink PSCs, as they do not require LIF and bFGF supplementation [37,38]. On the other hand, as mink PSCs were cultured on American mink or murine feeder cells, both growth factors may had been secreted in the medium in sufficient concentrations.

Recently, researchers have started to use particular small molecules to inhibit certain signal pathways that interfere with reprogramming. These inhibitors also may support pluripotency in some species or certain genotypes. The most widely used are 2i: MEK inhibitor PD0325901 and GSK3 inhibitor CHIR99021. Other popular small molecules include A 83-01, which is an inhibitor of TGF-β type I receptor ALK5 kinase, type I activin/nodal receptor ALK4, and type I nodal receptor ALK7, and forscolin, an activator of adenylyl cyclase (increases cAMF level). The other important molecules are ROCK kinase inhibitors that prevent cell death such as Y-27632 and thiazovivin. Human PSCs need one of these molecules to survive single-cell dissociation. PSCs could be passaged manually or enzymatically. The enzyme choice may be important for pluripotency maintenance. Signal pathway inhibitors were first tested on mouse and human PSCs; their usage in Carnivora PSC derivation may be beneficial, and some researchers have successfully used them (Table S1). On the other hand, our data show that 2i in “standard” concentrations is toxic for many carnivoran species fibroblasts, including fox and pinnipeds (Menzorov, Beklemisheva, unpublished). Recent research shows that the identification of small molecules for reprogramming facilitation may be automatized [51].

### 2.5. Matrix and Feeder Cells

Mouse and human PSCs were traditionally cultured on mitotically inactivated embryonic fibroblasts known as feeder cells. The majority of the researchers used mouse embryonic fibroblasts as feeder cells though some used species-specific autologous fibroblasts [14,37]. The feeder cells produce growth factors including LIF and bFGF and other molecules as well as an extracellular matrix whose function is to imitate a “normal” stem cell environment. Additionally, plastic culture plates are covered with a matrix that contains a mixture of different molecules such as gelatin or Engelbreth–Holm–Swarm mouse sarcoma matrix or with defined composition such as vitronectin or laminin-511. Some researchers used feeder cells for Carnivora PSC derivation but had not specified the usage of a matrix (Table S1); most probably, gelatin was used by default. Feeder-free PSC culture usually requires different matrices than the ones used with MEFs. Different matrices most probably differ in the efficiency of pluripotency maintenance. Kimura and colleagues showed that laminin-511 in combination with StemFit cell culture medium is superior to laminin-521 or Engelbreth–Holm–Swarm mouse sarcoma matrix with E8 or mTeSR1 media [20].

## 3. Analysis of Pluripotency

### 3.1. Key Pluripotency Genes

The first step in defining whether pluripotency had been established in the derived potential PSCs is testing for marker gene expression. The surface marker alkaline phosphatase (AP) is convenient, as its analysis is easy, and staining of live cells is available. As AP presence is measured based on its activity, species-specific differences between the proteins are not important. The disadvantage of AP is that it is not specific to PSCs and may not correspond to functional pluripotency. The role of AP in PSCs was reviewed by Štefková and colleagues [52]. Other widely used surface markers include SSEA-1, SSEA-3, SSEA-4, TRA-1-60, and TRA-1-81. The expression of these markers is species-specific. Usually, there is no information on whether the commercially available antibodies have cross-reactivity with carnivoran proteins, and it should be checked in advance if possible.

The core pluripotency gene network includes Oct4, Sox2, and Nanog [53]. Both human and mouse PSCs express these markers. The analysis of the Carnivora PSCs may be tricky, as *OCT4* and *SOX2* are often introduced to induce reprogramming and could be expressed exogenously. If human transgenes are used for reprogramming, the primers should be species-specific, and the same applies to the antibodies. It is necessary to analyze the expression of genes that are not introduced exogenously, such as *Nanog* (the majority of reprogramming “cocktails” does not include it), *Rex1*, and *Tert*. The main problem with all marker genes is that their expression is not sufficient for stating pluripotency. Pluripotency should be shown functionally as the ability of the supposed PSCs to differentiate into the cell types of all three germ layers.

### 3.2. Naïve and Primed Pluripotency

There are at least two types of pluripotency corresponding to different stages of embryo development—naïve and primed [54]. There are “permissive” mouse strains whose embryos readily transform into ES cells with naïve pluripotency: 129, BALB, and C57BL. It appeared that the signal pathways inhibition with 2i allows the generation of naïve ES cells from “non-permissive” genotypes [55]. Moreover, 2i and SU5402 were used to derive rat ES cells that were participating in the formation of the chimeric animals [56]. Human ES cells have primed pluripotency in standard culture conditions. Interestingly, it is possible to switch between naïve and primed states both for mouse and human PSCs (reviewed in [54]).

There are reports about additional pluripotency states [57]. Yu and coauthors were able to produce mouse, human, and horse PSCs with an intermediate between naïve and primed state: the “formative phase”. Basically, it means that for some species, it is possible to stop the developmental program at different stages. There are species-specific differences in gene expression between naïve and primed PSCs. Gafni and colleagues compared transcriptomes of the 29 naïve and primed human and mouse PSCs. Naïve mouse and human PSCs clustered together as well as primed mouse and human ones [58]. To the author’s knowledge, such a comprehensive analysis was not performed for the other species. There could be two related difficulties: species-specific gene expression patterns and the development of a universal framework for the bioinformatics analysis.

It is necessary to have single-cell transcriptome data of the species’ early-stage embryos at different stages to connect the PSC gene expression patterns with the real developmental stage. For example, Boroviak and colleagues compared single-cell transcriptomes of mouse, human, and marmoset embryos from zygote to late inner-cell mass stage and revealed both conserved and species-specific features [59]. After the derivation of single-cell transcriptome data for species-specific embryos, it would be possible to reliably place carnivoran PSCs onto the developmental landscape. The iPS cell derivation with OCT4, KLF4, SOX2, and MYC may produce different cell types [60]. Thus, single-cell transcriptome analysis allows the mapping of the reprogramming stages. In the case of successful PSC derivation, their status would depend on species-specific properties and cell culture conditions. To the author’s knowledge, there are no available single-cell transcriptome data for carnivoran PSCs.

Lack of a detailed molecular landscape of carnivoran species embryo development leads to an absence of a species-independent definition of PSC pluripotency states. Different researchers usually presume that obtained Carnivora PSCs have naïve or primed pluripotency based on morphology and expression level of several key genes. Morphologically, the majority of produced Carnivora PSCs are similar to human primed PSCs (Table S1). It is the author’s opinion that comparisons based on morphology and certain gene expression are generally not conclusive even for naïve and primed pluripotency, not to mention formative. Sometimes, even the morphology significantly differs. As an example, there are American mink PSCs that differ from mouse and human PSCs in morphology and gene expression [38]. Their status on the scale between human and mouse naïve and primed PSCs is unclear.

Generally, human iPS cells are readily produced from donor cells regardless of genotype. That is not the case for mouse PSCs [55]. Such genotype-specific efficiency of PSC derivation could be the case for the Carnivora PSCs. There were just a few cell lines of dog iPS cells produced in the majority of the published research (Table S1). Maybe part of the problem is that certain breed genotypes are “non-permissive”. Dog breeds are far from being as genetically similar as mouse strains, but the lack of information about the breed is a clear shortcoming of many reports.

### 3.3. X-chromosome Inactivation

One of the hallmarks of the transition from inner-cell mass to epiblast is X-chromosome inactivation. For human and mouse, PSC patterns of X-chromosome inactivation are well studied. Somehow, that is not the case for Carnivora PSCs. Just four reports paid attention to X-chromosome status; dog iPS cells and American mink ES cells had cells with both X-chromosomes in an active state [24,30,37,38]. We have also shown that both X-chromosomes are active in iPS cells of American mink, but the differentiation of these cells was not studied, so pluripotency was not shown [61].

### 3.4. Transgene Silencing

Reprogramming transcription factors are important during the shift of the developmental program from differentiated to pluripotent. Continuous transgene expression in iPS cells may lead to abnormal differentiation and malignization due to proto-oncogenes such as MYC. As PSCs silence retroviral expression, retro- or lentiviral vectors are generally silenced [43,44,45]. Sendai-virus-based vectors and episomes are usually designed in such a way that they are lost during prolonged cell culture. Due to the probability of reactivation of retroviral vectors and continuous expression of other types of vectors, it is necessary to show that exogenous transgenes are no more active. In Carnivora iPS cells, transgenes could be silenced or partially silenced, or their expression was not analyzed at all (Table S1). In the case of retro- and lentiviral vectors, it is not clear whether lack or partial silencing could be considered incomplete reprogramming. Furthermore, despite the lack of transgene silencing, cat iPS cells readily differentiated into derivatives of all three germ cell layers, thus demonstrating their pluripotency [34].

## 4. Cytogenetics

Mouse ES cells are prone to chromosome instability on the level of the chromosome number [62], and the same could be said about human PSCs [63]. Moreover, human PSCs tend to accumulate small genome rearrangements, leading to proto-oncogene accumulation [64]. Chromosome instability may influence pluripotency and differentiation potential due to disrupted gene expression regulation through mutations and gene dosage. Just 9 out of 27 reports about Carnivora PSCs paid attention to karyotype stability, and some provided no information about the karyotype, raising concerns about the species identity of the cells.

## 5. Differentiation of PSCs

### 5.1. Embryoid Bodies

Pluripotency could be defined as an ability to differentiate into the cell types that are representatives of all three germ layers. Optionally, PSCs may be able to differentiate into germ line cells [65,66]. Additionally, human PSCs are readily differentiated into trophoblast [67].

PCSs in certain culture conditions may be considered an embryo in an incorrect environment. They spontaneously differentiate into a mixture of cells that belong to three germ layers. These cell agglomerates are called embryoid bodies (EBs). The presence of marker genes of three germ layer cell types may be considered a minimal requirement to state pluripotency. An alternative option is to perform directed differentiation of PSCs with or without the EB stage into the different cell types. Usually, differentiation protocols for Carnivora PSCs are adapted from the mouse and human ones.

It is important to note that the presence of pluripotency marker genes in potential PSCs does not signify pluripotency. Only functional analysis, i.e., differentiation, allows the demonstration of pluripotency.

### 5.2. Teratomas

Teratoma-formation assay in immunodeficient mice is sometimes considered the gold standard for pluripotency demonstration, at least for human PSCs [68]. PSC differentiation in teratomas takes longer than in EBs, and there are many highly differentiated cell types. On the other hand, even for human and mouse PSCs, the teratoma assay is not standardized. The main problems are inconsistency in the teratoma derivation methodology and the absence of a standardized teratoma analysis report [69].

The situation is worse for the PSCs that are not of human or mouse origin. Only 12 reports demonstrated the derivation of teratomas with cells from all three germ layers, some were unable to produce teratomas, and some groups had not tried (Table S1). There are many protocols for teratoma derivation. Injected cell number, site of the injection, co-injection with matrix or feeder cells, and recipient genotype greatly vary and may influence the result. It is unclear how to standardize such a test. For instance, among teratomas produced from American mink MES29 ES cells, histological analysis revealed that only two out of nine contained cell types from all three germ layers. The remaining seven teratomas lacked either ectodermal, endodermal, or both cell derivatives [38]. If there were fewer teratomas, we may have presumed that these ES cells were not pluripotent. Thus, if PSCs can give rise to cell derivatives of all three germ layers in teratomas, that proves pluripotency. A negative result shall not be considered as concluding.

### 5.3. Chimeras, Tetraploid Complementation, and Germ-Line Transmission

The “ultimate” test of pluripotency is the ability of PSC derivatives to participate in normal development. Mouse PSCs can form chimeric animals when inserted into the blastocyst or mixed with early-stage embryos. Such chimeras may produce gametes with the donor genotype. Moreover, in the combination with a tetraploid embryo, mouse PSCs can support full development [70]. Recently, tetraploid complementation was shown for rat ES cells [71]. Such achievements remain theoretical for Carnivora PSCs due to a lack of knowledge of their embryonic development and technical difficulties.

## 6. Prospects and Recommendations

There is a limited number of reports of carnivoran species’ iPS cell generation. It seems highly likely that cell culture conditions for pluripotency induction and maintenance differ between species. The genotype of the animals may also play a role, at least for some species. The regulation of pluripotency may have species-specific features.

Two approaches to PSC derivation experiment design for the novel species could be employed. The “fundamental” approach may rely on the study of the biology of the species, gathering data on gene expression and signal pathways in the embryonic cells. Another approach is “practical”. There are protocols for PSC derivation for multiple species readily available. The combination of the protocols’ parts may give a result. For instance, it appears that for pluripotency maintenance in the carnivoran species PCSs, either LIF, LIF and bFGF, or bFGF alone are necessary. The same applies to the other protocol specifics (Figure 1).

The efficiency of iPSC derivation is extremely low for the majority of the protocols. One of the reasons may be that during cell reprogramming, there are different cell fate trajectories, and pluripotent cell appearance is represented by just a minor probability [60]. Several factors that should be considered for iPS cell derivation from a novel species from a technical point of view: (a) transgene delivery method, (b) the choice of a transgene set, and (c) cell culture conditions.

Minimal characterization of the generated iPS cells shall include pluripotency marker expression analysis, differentiation into three germ layer cell types by EBs and directed differentiation, transgene silencing analysis, and cytogenetic analysis. In the author’s opinion, the cytogenetic analysis should include the study of the karyotype stability. In addition, X-inactivation analysis should be performed for female cells. The teratoma-formation pluripotency test may be performed to glimpse into the ability of the cells to differentiate into the “adult” cell types. Transcriptome and methylome analysis would be an important contribution to the field. With the accumulation of publicly available data on gene expression, it may be possible in the future to understand the evolution of pluripotency [72] and even to suggest ways of more efficient pluripotency induction.

## Supplementary Materials

The following supporting information can be downloaded at: https://www.mdpi.com/article/10.3390/ijms24043905/s1.
